# RESVECH 2.0: cross-cultural adaptation for Brazil, reliability and validity for the evaluation of venous ulcers

**DOI:** 10.1590/0034-7167-2022-0185

**Published:** 2023-03-27

**Authors:** Marina Rosa Menegon, Suelen Gomes Malaquias, Joana Aragão da Silva, Beatriz Guitton Renaud Baptista de Oliveira, Juan Carlos Restrepo Medrano, José Verdú-Soriano, Maria Márcia Bachion

**Affiliations:** I Universidade Federal de Goiás. Goiânia, Goiás, Brazil; II Universidade Federal Fluminense. Niterói, Rio de Janeiro, Brazil; III Productivity Fellow of the National Council for Scientific and Technological Development - CNPq. Brasília, Distrito Federal, Brazil; IV Universidad de Antioquia. Medellin, Colombia; V Universidad de Alicante. San Vicente del Raspeig, Alicante, Spain

**Keywords:** Varicose Ulcer, Healing, Nursing Assessment, Cross-Cultural Comparison, Psychometrics., Úlcera Varicosa, Cicatrización de Heridas, Evaluación en Enfermería, Comparación Transcultural, Psicometría., Úlcera Varicosa, Cicatrização, Avaliação em Enfermagem, Comparação Transcultural, Psicometria.

## Abstract

**Objectives:**

to cross-culturally adapt the scale *Resultados en la valoración y evolución de la cicatrización de las heridas* - RESVECH 2.0 for Brazilian Portuguese; to estimate the internal consistency and construct and criterion validity of the scale in the evaluation of venous ulcers.

**Methods:**

methodological study, based on international guidelines for studies of this type. Wounds were evaluated using the RESVECH 2.0 and Pressure Ulcer Scale of Healing 3.0 (PUSH). Descriptive analysis, confirmatory factor analysis, Cronbach’s alpha and Spearman’s correlation (p<0.05) were used.

**Results:**

12 nurses and 77 people with 153 venous ulcers participated in the study. The translation was successful, the proposed factor model was validated, and Cronbach ‘s alpha = 0.832 (95%CI, 0.780-0.880) and correlation coefficient (RESVECH 2.0 and PUSH 3.0) = 0.74 were obtained.

**Conclusions:**

the adaptation of RESVECH 2.0 to Brazilian Portuguese is robust. Reliability and validity show compatibility for use in the country in the evaluation of venous ulcers.

## RESUMO

**Objetivos:**

adaptar transculturalmente a escala *Resultados en la valoración y evolución de la cicatrización de las heridas* - RESVECH 2.0 para o português do Brasil; estimar sua consistência interna, validade de construto e de critério para utilização em úlceras venosas.

**Métodos:**

estudo metodológico, baseado em diretrizes internacionais para estudos dessa natureza. Realizou-se avaliação das feridas por meio da RESVECH 2.0 e da *Pressure Ulcer Scale of Healing* 3.0 (PUSH). Empregou-se análise descritiva, análise fatorial confirmatória, alfa de Cronbach e correlação de Spearman (p<0,05).

**Resultados:**

participaram 12 enfermeiros e 77 pessoas com 153 úlceras venosas. A tradução foi bem-sucedida, o modelo fatorial proposto foi validado, obteve-se alfa de Cronbach = 0,832 (IC95%=0,780-0,880) e coeficiente de correlação (RESVECH 2.0 e PUSH 3.0) = 0,74.

**Conclusões:**

a adaptação da RESVECH 2.0 para o português do Brasil é robusta. A confiabilidade e validade evidenciam compatibilidade para utilização no país e avaliação de úlceras venosas.

## RESUMEN

**Objetivos:**

adaptar transculturalmente la escala “Resultados en la valoración y evolución de la cicatrización de heridas”, RESVECH 2.0 al portugués de Brasil; estimar su consistencia interna, validez de constructo y de criterio para su utilización en úlceras varicosas.

**Métodos:**

es un estudio metodológico, basado en directivas internacionales sobre investigaciones de esta naturaleza. Se evaluaron las heridas por medio de la RESVECH 2.0 y de la Escala de Cicatrización de Úlceras por Presión 3.0 (PUSH). Se llevó a cabo con análisis descriptivo, análisis factorial confirmatorio, alfa de Cronbach y correlación de Spearman (p<0,05).

**Resultados:**

participaron 12 enfermeros y 77 personas que tenían 153 úlceras venosas. La traducción fue exitosa, el modelo factorial propuesto fue validado, el alfa de Cronbach = 0,832 (95%CI=0,780-0,880) y el coeficiente de correlación (RESVECH 2.0 y PUSH 3.0) = 0,74.

**Conclusiones:**

la adaptación de la RESVECH 2.0 al portugués brasileño es sólida. La fiabilidad y la validez demuestran la compatibilidad para su utilización en el país en la evaluación de las úlceras varicosas.

## INTRODUCTION

Venous ulcers (VUs) are the most common type of leg ulcer and are usually associated with chronic venous insufficiency^(1-2)^. This type of injury often appears in the leg, between the knee and the ankle, but sometimes it also appears in areas below the ankle^(3)^. It usually heals slowly^(1-3)^ and has a high chance of relapse^(4)^.

Seeing that these injuries are chronic and are monitored by a multidisciplinary team in the primary and secondary care of the Unified National Health System, it is essential to use scales to assess the evolution of healing, in order to allow standardized clinical evaluation records, ensure effective communication among professionals, provide an accurate assessment of the outcomes of the care provided and help the decision-making process regarding the care techniques to be used.

There are dozens of instruments to assess wound healing^(5-7)^. Most of them are focused on pressure injuries or chronic wounds in general, but very few are aimed at the evaluation of leg ulcers, and none address venous ulcers specifically. Despite the variety of scales available, no instrument is considered the gold standard to assess every type of wound, including venous ulcers.

The Pressure Ulcer Scale for Healing (PUSH)^(8-9)^ is one of the most widely used and recognized scales in the world^(10)^. Originally developed to assess the healing of pressure injuries, it has since been used to assess other types of chronic wounds, such as leg ulcers in general^(11-12)^ and venous ulcers^(13-15)^, in several countries ^(12,15-16)^, including Brazil^(11,15)^. The scale has been cross-culturally adapted to Brazilian Portuguese^(17)^ and has a very good inter-rater reliability in the evaluation of leg ulcers^(11)^ and venous ulcers^(15)^ and good responsiveness in chronic wounds (pressure injuries, neuropathic ulcers and venous ulcers)^(18)^.

However, the characteristics of venous ulcers are not fully considered in the PUSH assessment parameters, as these injuries can affect extensive areas, result in infection^(14,19)^, and be painful^(19-20)^.

In an attempt to develop a more comprehensive instrument to evaluate chronic wounds, researchers from the *Grupo Nacional para el Estudio y Asesoramiento en Úlceras por Presión y Heridas Crónicas* (GNEAUPP) in Spain developed the *Resultados Esperados de la Cicatrización de las Heridas Crônicas* (RESVECH) scale^(5,21)^. The version 1.0 of the scale contained nine items; however, after the first clinical validation tests, three of them were excluded (periwound maceration, tunneling and pain), resulting in version 2.0^(21)^. Items currently evaluated include: dimensions of lesion; depth and tissues involved; edge features; tissues in the wound bed; exudate; and signs of infection/inflammation^(21-22)^, which are relevant for the evaluation of venous ulcers^(19-20)^. The final assessment is based on the score obtained, which can range from 0 (healed wound) to 35 points (worst possible condition)^(22)^. The scale has been cross-culturally adapted for use in other countries such as Colombia^(23)^, Portugal^(24)^ and Brazil^(25)^.

The first translation and cross-cultural adaptation into Brazilian Portuguese was carried out in a city in the state Minas Gerais^(25)^ and resulted in changes in the scale, without showing whether this occurred with the author’s consent. In that research, there was no testing of psychometric properties when applied to the population.

Valid and reliable instruments can contribute to clinical practice, health assessment and research, support decision-making^(25-26)^, favor the assessment of the healing process and contribute to a standardized and effective communication of the results observed during and at the end of the treatment of people with VU.

Thus, the cross-cultural adaptation of the RESVECH 2.0 in a broader context and the verification of the reliability of the Brazilian version are essential to provide better evidence to support its use in the evaluation of people with venous ulcers in Brazil.

The results of this study are intended to contribute to the clinical performance of nursing and health professionals, through a more accurate assessment of the wound healing process, allowing the analysis of the effectiveness of treatments and supporting professional nursing practice and evidence-based health care. Additionally, this study may contribute to teaching in the area of evaluation and treatment of wounds in undergraduate and *lato sensu* graduate programs, and enable the use of the scale in a protocol for the evaluation of healing of venous ulcers in future research.

## OBJECTIVES

To cross-culturally adapt the scale *Resultados Esperados de la Valoración y Evaluación de la Cicatrización de las Heridas Crônicas* (RESVECH 2.0) into a Brazilian Portuguese version.

To estimate the internal consistency and construct and criterion validity of the Brazilian version of RESVECH 2.0 in the evaluation of venous ulcers.

## METHODS

### Ethical aspects

This is an excerpt from a matrix project called “*Tradução, adaptação transcultural, confiabilidade e responsividade de escalas de avaliação de capacidade funcional, cicatrização e qualidade de vida de pessoas com úlceras venosas*”, funded by CNPq [Process 312093/2013-6]. The study follows the recommendations of Resolution No. 466 of 2012 of the National Health Council^(27)^ and was approved by the Research Ethics Committee of the Hospital das Clínicas of the Federal University of Goiás.

### Type of study

Methodological multicenter study carried out in Goiânia-GO and Niterói-RJ, from 2016 to 2018.

### Cross-Cultural Adaptation

**Figure 1:**
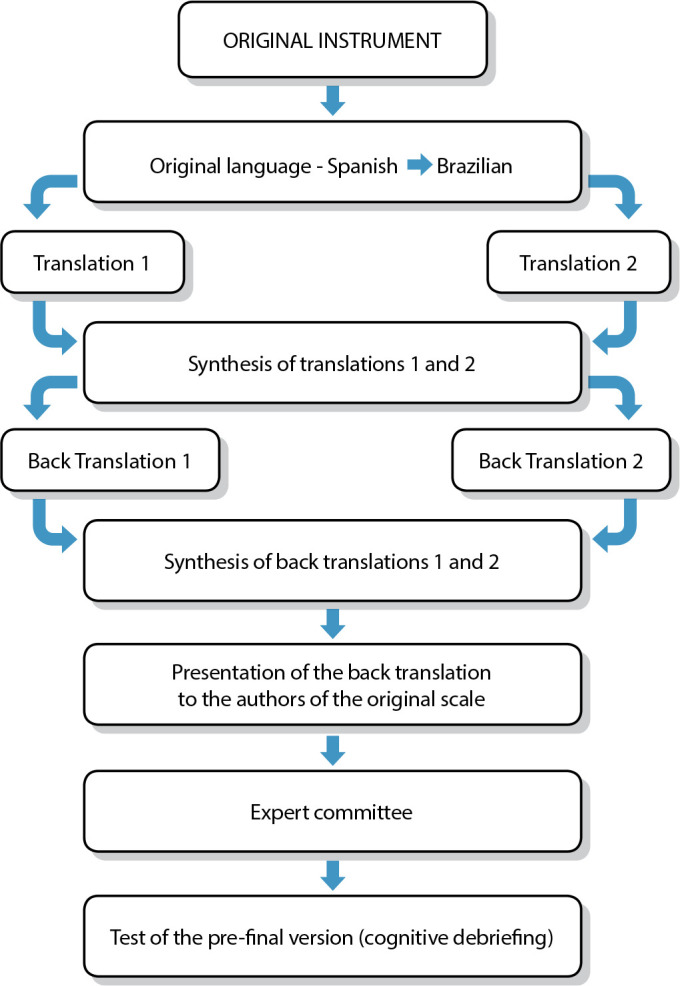
Flowchart of the translation process

The recommendations from the literature on translation and cross-cultural adaptation were followed in the process^(28-30)^. Thus, the steps shown in Figure 1 were followed:

### Data collection

Data was collected from 2016 to 2018, in Goiânia-GO and Niterói-RJ, in two stages.

The cognitive debriefing of the pre-final version (translated) was conducted with nurses with experience in the treatment of people with venous ulcers, according to the recommendations in the literature^(29-31)^. Professionals were invited to fill out a characterization form and carefully analyze the translated instrument, recording any doubts.

The analysis of the psychometric properties was conducted with people with venous ulcers undergoing outpatient treatment in the study settings. The following inclusion criteria were applied: age ≥ 18 years; satisfactory score in the mini-mental state examination^(32)^, according to the level of education; medical diagnosis of chronic venous insufficiency; presence of clinical signs of venous insufficiency; active venous ulcer. Those with signs of moderate or severe arterial impairment were excluded.

After receiving training, the researchers applied the translated version of RESVECH 2.0 and PUSH 3.0 in people with venous ulcers. This scale was chosen as reference because it is used worldwide and has acceptable psychometric properties for use in this population, as described above.

### Data analysis

**Figure 2:**
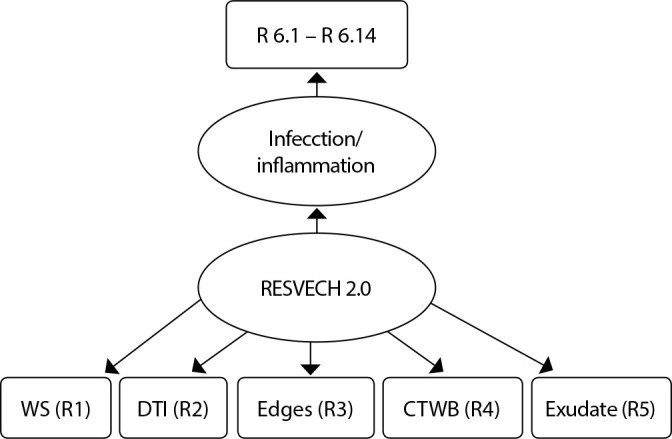
Factorial structure to be tested for the validation of the scale *Resultados en la valoración y evolución de la cicatrización de las heridas* - RESVECH 2.0

The collected data were organized in an electronic database using the software Statistical Package for the Social Sciences (SPSS). For construct (or concept) validation, confirmatory factor analysis (CFA) was used to evaluate the factor structure of the RESVECH 2.0 with 19 items (Figure 2). Dimensions 1 to 5 consist of isolated items and dimension 6 (inflammation/infection) includes 14 items. CFA was chosen because it is considered the most appropriate method for evaluating dichotomous variables and minimizing the risks of high standard error in the correlation coefficients, allowing the grouping of items that are associated with each other and determining the relationship between a group of variables^(33)^.

*DL - Dimensions of the lesion; DTI - Depth/ tissues involved; TWB -Tissues in the wound bed; Exudate; R - item of the RESVECH scale.*

Initially, the data was tested to verify the adequacy of the CFA model, using a matrix of correlation coefficients with the 19 items to assess the degree of correlation between them. A tetrachoric correlation matrix was used to verify the correlation between the nominal items and the the rank-biserial correlation coefficient^(34)^ with p-value<0.05 was used to verify the correlation between ordinal and nominal, and ordinal and ordinal items. Then, the Bartlett test of sphericity^(35)^ and Kaiser-Meyer-Olkin (KMO) test were performed to verify the adequacy of the CFA model. For the Bartlett test, a p-value<0.05 was adopted. In turn, in the KMO test, a result above 0.600 was considered adequate^(36)^. The model’s Cronbach’s alpha was also calculated, considering values>0.78 as acceptable^(31,37)^. Spearman’s correlation coefficient (r_s_) was used to analyze the validity related to the criterion between the data obtained from RESVECH 2.0 and from PUSH 3.0. A value of p<0.05 was adopted. Values above 0.70 were considered acceptable^(37)^.

## RESULTS

### Cross-cultural adaptation of RESVECH 2.0 to Brazilian Portuguese

There were few difficulties in the translation of the RESVECH 2.0 scale, and, as expected, they occurred on the part of the professional that was not from the health area, who used, for example, the term *escara* to refer to ulcer. In the stages of expert committee analysis and testing of the pre-final version, some terms were discussed as, even though they exist in Brazilian Portuguese, they are not common in the research scenario or do not represent phenomena for which there is more current terminology in clinical practice.

A total of 12 nurses participated in the cognitive debriefing stage. The participants considered that the use of the term “*danificadas*” to refer to the conditions of the edges was odd. After discussion of semantics (*danificadas* = that which has been flawed or harmed; *lesada* or *deteriorada* = changed for the worse; damaged, according to *Oxford Languages*) and considerations about the most common term in clinical practice, the term “*deteriorada*” was chosen to refer to the wound edges.

Another term discussed in the context of evaluation of wound edges, was “*bordas engrossadas (envelhecidas ou evertidas)*”. After discussion with the authors of the original scale, these terms were maintained and more details were provided in the description of these items on the guiding instrument for application of the scale (Appendix 1 - Supplementary material). Still regarding the evaluation of wound edges, the cognitive debriefing participants found it difficult to understand the difference between “*bordas não distinguíveis (não há bordas)”* and *“bordas não delimitadas”*, or *“bordas não distinguíveis x fechada/cicatrizada”*. Upon clarification based on stage I pressure ulcers, in which there is no rupture of the epidermis, and therefore no wound edges, the participants understood the use of the expression in the context, but highlighted that, in the case of venous ulcers, the category *“bordas não distinguíveis”* would not be applicable.

Exudate was evaluated in the RESVESCH 2.0 based on the terms *“seco”, “úmido”, “molhado”, ”saturado”* and *“com fuga de exsudato”*. Likewise, although the terms are understandable, they are not common in clinical practice in the setting of the present investigation, where the professionals usually assess the exudate as “*ausente”, “pequeno”, “moderado”* or “*grande”*. The authors of the original scale did not authorize changes in the options for the evaluation of exudate. They consider that, as in other scales, adequate training must be provided so that the instrument is perceived in its scope as originally envisioned.

There were no issues with the item *“tecido compatível com biofilme”*, but later, in the researchers’ training, further clarification was required for the standardized evaluation of this item. Thus, the following description was added to guide the assessment of the presence of tissue compatible with biofilm: *tejido compatível con biofilm es una capa de “sustância” sobre la ferida de color blanco-amarillento, ou transparente pero brillante (habitualmente clasificado como esfacelo o como fibrina, pero que en este caso se retira fácilmente con una torunda o gasa)* (José Verdu Soriano).

All other items of the scale were successfully translated, with no issues in semantics or cultural understanding, resulting in a culturally adapted version of the RESVECH 2.0 (Appendix 2 - Supplementary material) for further analysis of internal consistency.

To assess internal consistency, 153 VUs presented by 77 participants were evaluated. Of these, 36 were linked to the research center of UFF and recruited in the referral outpatient clinic for wound care in Niterói-RJ, and 41 were linked to the UFG research center and recruited in the outpatient wound care network in Goiânia-GO.

Among the participants, 42 (54.5%) were female and 35 (45.5%) were male. Approximately half (54.9%) of the lesions had an area equal to or greater than 24 cm^2^ and 19% had an area equal to or greater than 100 cm^2^.

### Confirmatory Factor Analysis

**Figure 3:**
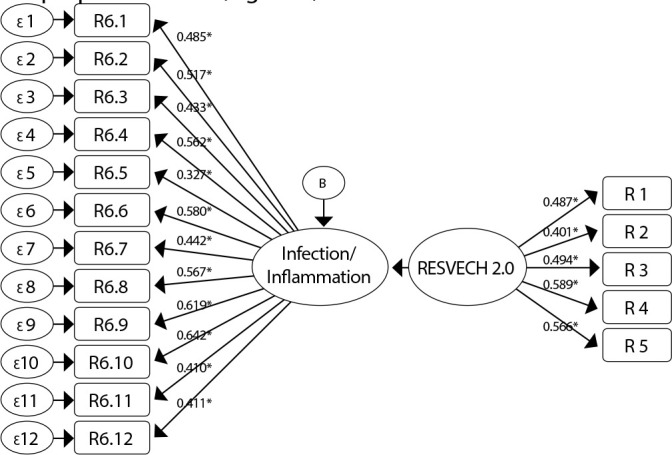
Path diagram of the factorial structure of the scale *Resultados en la valoración y evolución de la cicatrización de las heridas* - RESVECH 2.0 validated with 19 items

The KMO test value was 0.615, demonstrating the suitability of data for the CFA. Also, the probability of Bartlett’s test of sphericity suggested the factorability of the correlation matrix (chi-square: 648.006; p-value<0.001)^(35)^. Confirmatory factor analysis showed that most items had a significant correlation coefficient >0.3 (Table 1), indicating the factorability of the matrix, according to the proposed model (Figure 3).

**Table 1:** Results of the Confirmatory Factor Analysis of the scale *Resultados en la valoración y evolución de la cicatrización de las heridas* - RESVECH 2.0

Variable	β	95%CI	Standard Error	*p* value	FL
Item					
1 Dimensions of the lesion	0.687	0.513 - 0.862	0.089	<0.001	0.487
2 Depth/tissues involved	0.307	0.119 - 0.406	0.096	0.001	0.401
3 Edges	0.303	0.112 - 0.494	0.097	0.002	0.494
4 Slough	0.249	0.050 - 0.446	0.100	0.014	0.589
5 Exudate	0.578	0.402 - 0.752	0.089	<0.001	0.566
6. Inflammation/Infection					
6.1 Increased pain	0.157	0.032 - 0.346	0.096	0.045	0.485
6.2 Perilesional erythema	0.515	0.370 - 0.659	0.073	<0.001	0.517
6.3 Perilesional edema	0.209	0.025 - 0.396	0.094	0.026	0.433
6.4 Increased temperature	0.652	0.512 -0.703	0.072	<0.001	0.562
6.5 Increased exudate	0.128	0.057 - 0.313	0.094	0.048	0.327
6.6 Purulent exudate	0.372	0.188 - 0.556	0.094	<0.001	0.580
6.7 Friable tissue	0.204	0.187 - 0.206	0.100	0.924	0.442
6.8 Stagnant wound	0.609	0.450 - 0.766	0.079	<0.001	0.567
6.9 Biofilm compatible tissue	0.408	0.306 -0.655	0.088	<0.001	0.619
6.10 Odor	0.567	0.407 - 0.728	0.082	<0.001	0.642
6.11 Hypergranulation	0.203	0.017 - 0.422	0.112	0.041	0.410
6.12 Size increase	0.489	0.294 - 0.683	0.099	<0.001	0.411
6.13 Satellite injuries	0.208	0.029 - 0.386	0.091	0.023	0.386
6.14 Paleness of the tissue	0.147	0.067 - 0.273	0.107	0.042	0.321

*β - Regression coefficient; 95%CI - 95% Confidence Interval; FL - Factor loading.*

ɛ/B - Standard error; * - Factor loading.

All factor loadings had values > 0.4 (Table 1), except for item 6.5, items 6.13 and 6.14. However, these items still presented acceptable factor loading values ( > 0.3).

### Internal consistency of the translated version of RESVECH 2.0

Internal consistency was estimated using Cronbach’s alpha with a 95% confidence interval. The proposed factorial model showed that the estimated internal consistency of REVESH 2.0 was 0.832 (95% CI = 0.780-0.880), indicating good internal reliability.

### Criterion-related validity of the translated version of RESVECH 2.0

A correlation coefficient of 0.74 supported the criterion validity of the scale. It was found that the items *“dimensão”, “tecido”, “exsudato”*, total RESVESCH 2.0 score and respective PUSH items presented a strong (r_s_ >0.70) correlation with each other (Table 2). As for the other items of the RESVECH 2.0, it is worth noting that the size of the lesion showed a moderate correlation (r_s_ 0.40 to 0.69) with other items of the PUSH 3.0.

**Table 2:** Spearman’s correlation coefficient between the items of the scales *Resultados en la valoración y evolución de la cicatrización de las heridas* - RESVECH 2.0 and *Pressure Ulcer Scale of Healing -* PUSH 3.0

REVESCH 2.0	PUSH 3.0			
	Area	Exudate	Tissue	Total
Dimensions of the lesion(r_s_)	0.892	0.609	0.121	0.878
p value	**<0.001**	**<0.001**	0.135	**<0.001**
Depth/ tissues involved (r_s_)	0.243	0.236	0.120	0.257
p value	**0.003**	**0.003**	0.139	**0.001**
Edges (r_s_)	0.174	0.270	0.106	0.254
p value	**<0.001**	**0.001**	0.193	**0.002**
Slough (r_s_)	0.087	0.138	0.789	0.204
p value	0.284	0.090	**<0.001**	**0.011**
Exudate (r_s_)	0.402	0.624	0.186	0.536
p value	**<0.001**	**<0.001**	0.021	**<0.001**
Item 6 (r_s_)	0.325	0.327	0.137	0.331
p value	**<0.001**	**<0.001**	0.069	**<0.001**
Total (r_s_)	0.683	0.641	0.247	0.740
p value	<0.001	<0.001	**0.002**	**<0.001**

*r_s_ - Spearman’s coefficient*

## DISCUSSION

The translation of RESVECH 2.0 was successful and no major difficulties were found in the process. Ease of translation for other contexts has also been reported^(23-25)^.

The fact that some terms were considered odd for use in clinical practice in Brazil may be due to the specificity of the present investigation, which addressed only the context of VU assessment.

This version of the instrument is robust, as the principles of good practices were followed^(28-30)^, and represents a relevant resource for clinical practice in Brazil, as there is evidence it can be applied in VU treatment, enabling the integration of signs and symptoms of infection, which are of great relevance for monitoring the healing process of these wounds.

It should be noted that the RESVECH 2.0 allows recording the size(area) of wounds up to 100 cm^2(5)^. This is important data, considering that VUs usually have areas larger than 24 cm^2(20)^, as considered in PUSH and corroborated in the present study.

Two items of the second category “Depth/tissues affected” would not be applicable in the context of VU: “involvement of the muscle”; “involvement of bones and/or surrounding tissue”, as most VUs are superficial^(20,38)^. However, this is not a limiting factor for the use of the scale in this population.

In other settings, the applicability of the term “*borda*” in clinical practice was not questioned, but in another region of Brazil, where the first RESVECH 2.0 translation was carried out, the term was changed to “*margem*”, which, according to the authors^(25)^, was more common in Brazilian culture, which was not confirmed in the present study.

It is more common to describe the edges as: *“epitelizadas”, “maceradas”, “com hiperceratose”, “hiperemiadas”* and/or *“com crostas”* ^(39)^, and these options generally apply to venous ulcers. Perhaps that is why nurses were surprised when seeing the option “*bordas engrossadas (envelhecidas ou evertidas)*”. As previously highlighted, it is expected that adequate training will be sufficient to overcome any difficulties in the understanding of the clinical phenomena to which the items refer.

There was no difficulty in translating or understanding the items in the category “type of tissue in the wound bed”. Most of the options available meet the characteristics of venous ulcers, in which slough is usually present^(20)^, as venous hypertension reduces blood flow in the capillary network, triggering a decrease in oxygen circulation, causing adhered neutrophils to activate and release free radicals and chemotactic substances, which damage the tissue, leading to tissue death^(40)^.

In other studies that translated the REVECH 2.0, there was also discussion regarding the description of exudate^(23-24)^, as occurred among the nurses participating in the present study. In the first translation to Brazilian Portuguese, the description of this item was changed to *“pequena”, “média”* and *“grande quantidade”*, according to the opinion of the experts^(25)^.

In the description of the item exudate in RESVECH 2.0, the present translation resulted in the terms: *“seco”, “úmido”, “molhado”, “saturado”* and *“com fuga de exsudato”*, as the authors did not authorize the alteration of the response options. This set provides more options, allowing a more refined assessment of the evolution of the amount of exudate. This means that a small change in the amount of exudate could be observed through RESVECH 2.0 when one evaluation indicates *“com fuga de exsudato”* and the subsequent one indicates *“saturado”*. Both options would be reported as *“grande quantidade”* if using the PUSH. The evaluation and description of the saturation of dressings can be an advantage, since, when compared to the PUSH, it admits broader answers for situations of high exuding wounds. In VUs, this may occur due to prolonged periods with legs down, low adherence to compression therapy and congestive heart failure, phenomena related to increased capillary permeability and osmotic hydrostatic pressure^(41)^.

The category “infection/inflammations” has 14 items, including pain assessment, allowing a refined evaluation^(3,42)^. It includes aspects commonly observed in the care of people with venous ulcers, therefore, no difficulties in understanding were mentioned in the *cognitive debriefing.*

The analysis of the factor model indicates the validity of the construct, with aspects 1 to 5 consisting of isolated items and aspect 6 “infection/inflammation” encompassing 14 items. Although item 2 (“depth/tissues involved”) had a loading factor within the acceptable limit, little correlation was observed with the other items on the scale. This can be explained by the fact that, as verified in the present study and mentioned in other studies^(20,41)^, most VUs are superficial, with involvement of the subcutaneous tissue. It is a predominant, relatively stable condition, whose evolution would be epithelialization itself. In this sense, although clinically relevant, the assessment of this item remains stable, while other items, such as the wound area, evolve substantially, contributing to the absent correlation in the case of venous ulcers.

The REVECH 2.0 depth evaluation includes an option that is not very applicable to venous ulcers, the destruction of tissues reaching the muscles, tendons, or bones. It is a condition that can occur in pressure injuries, or in arterial or mixed ulcers, but is not common in VUs^(20,41)^.

In turn, the presence of necrotic/devitalized tissue, especially slough, is a predominant characteristic of VUs, as shown in this and other studies^(22,41,43)^, which may explain the fact that, despite presenting a high factor loading (0.589), item 4 “type of tissue in the wound bed” had little correlation with the other items on the scale.

In the domain 6 of the scale, items 6.5, 6.13 and 6.14 (“increasing exudate”, “satellite lesions” and “paleness of the tissue do tecido” respectively), presented lower factor loadings, indicating that they may not be significantly associated with the other items of the scale^(33)^ considering people with venous ulcers, who are the specific population studied.

The International Wound Infection Institute^(42)^ indicates the following signs/symptoms infection, based on consensus: erythema, local warmth, edema, purulent discharged, delayed wound healing, increasing pain, increasing malodor. This data corroborates the findings of the present study, which showed that these items have significant factor loadings, demonstrating their relevance for wound assessment and clinical decision-making. In the case of venous ulcers, wound infections must be managed^(42)^, as they can reduce the chance of healing by up to 42%^(44)^ and lead to new or increasing pain^(45)^.

The Cronbach’s alpha coefficient in the factor analysis model indicated that the internal consistency of the Brazilian Portuguese version of RESVECH 2.0 was 0.832. This result cannot be compared to Cronbach’s alpha coefficients obtained in studies that did not use a factorial model. Having made this observation, the value obtained in the present study meets the recommended values, that is, above 0.70^(37)^, and is within the 95% confidence interval (95%CI = 0.780-0.880), indicating good internal consistency and demonstrating the cohesion and coherence of the items to assess the construct validity of the RESVECH 2.0, that is, the conditions of the healing process in VU cases.

The criterion validity was supported by a coefficient of 0.74 between RESVECH 2.0 and PUSH 3.0, indicating a strong correlation and reiterating the relevance of RESVECH 2.0 - adapted version for clinical practice in the care of people with chronic wounds, which include venous ulcers.

Considering that care for people with venous ulcers is predominantly provided in the outpatient care network, in primary care, where staff turnover is high, the use of a reliable and standardized instrument to assess the healing of these lesions can contribute to effective communication between professionals, supporting the evaluation of the outcomes of the care provided and the decision-making process regarding the care technologies to be used.

### Study limitations

A limitation of this study was that it was carried out in only two setting, in a country of continental size such as Brazil. However, the fact that it was carried out in more than one region of the country is already an advance.

### Contributions to the Area

Considering that care for people with venous ulcers is predominantly provided in the outpatient care network, in primary care, where staff turnover is high, the use of a reliable and standardized instrument to assess the healing of these lesions can contribute to effective communication between professionals, supporting the evaluation of the outcomes of the care provided and the decision-making process regarding the care technologies to be used.

## CONCLUSIONS

A robust adaptation of the instrument to Brazilian Portuguese, with good reliability (internal consistency) and criterion-related validity was produced. The scale had appropriate psychometric properties for use in the country in the evaluation of venous ulcers. It will allow the evaluation of lesions with areas larger than 24 cm^2^ and the integration of signs and symptoms of infection and pain assessment, which is of great clinical relevance for monitoring the healing of this type of wounds.

## Data Availability

https://doi.org/10.48331/scielodata.RCMAU5
